# Pro-apoptotic Bid is required for the resolution of the effector phase of inflammatory arthritis

**DOI:** 10.1186/ar2204

**Published:** 2007-05-17

**Authors:** John C Scatizzi, Jack Hutcheson, Emily Bickel, G Kenneth Haines, Harris Perlman

**Affiliations:** 1 Saint Louis University, School of Medicine, Department of Molecular Microbiology and Immunology, Saint Louis, MO 63104, USA; 2 Yale University, School of Medicine, Department of Pathology, New Haven CT 06510, USA

## Abstract

Rheumatoid arthritis is an autoimmune disease characterized by hyperplasia of the synovial lining and destruction of cartilage and bone. Recent studies have suggested that a lack of apoptosis contributes to the hyperplasia of the synovial lining and to the failure in eliminating autoreactive cells. Mice lacking Fas or Bim, two pro-apoptotic proteins that mediate the extrinsic and intrinsic death cascades, respectively, develop enhanced K/BxN serum transfer-induced arthritis. Since the pro-apoptotic protein Bid functions as an intermediate between the extrinsic and intrinsic apoptotic pathways, we examined the role that it plays in inflammatory arthritis. Mice deficient in Bid (Bid-/-) show a delay in the resolution of K/BxN serum transfer-induced arthritis. Bid-/- mice display increased inflammation, bone destruction, and pannus formation compared to wild-type mice. Furthermore, Bid-/- mice have elevated levels of CXC chemokine and IL-1β in serum, which are associated with more inflammatory cells throughout the arthritic joint. In addition, there are fewer apoptotic cells in the synovium of Bid-/- compared to Wt mice. These data suggest that extrinsic and intrinsic apoptotic pathways cooperate through Bid to limit development of inflammatory arthritis.

## Introduction

Rheumatoid arthritis (RA) is an autoimmune disease characterized by hyperplasia of the synovial lining, inflammation, high levels of circulating and local IL-1β and tumor necrosis factor (TNF)α, and destruction of cartilage and bone. Antagonists to IL-1β or TNFα lead to decreased joint destruction through an unknown mechanism and also result in reduced numbers of macrophages [1], one of the principal cell types that contribute to the pathogenesis of RA. Since the numbers of macrophages are associated with worse clinical outcome [2,3], one prevailing hypothesis is that there is a failure to delete autoreactive cells, particularly macrophages, in the RA joint [4]. While the RA joint is replete with noxious molecules, including reactive oxidative species and death ligand expressing cells, histological evidence of apoptosis is rarely observed [5,6]. The induction of synoviocyte apoptosis in animal models of inflammatory arthritis results in either amelioration of the disease or reduction in joint inflammation and destruction [7-9]. Additionally, patients with pauciarticular juvenile chronic arthritis display enhanced mononuclear cell apoptosis in synovial tissue compared to patients with polyarticular arthritis [10]. These data suggest that increasing the level of apoptosis in the joint may be associated with improved clinical outcome. However, the apoptotic factors that are essential to limit the inflammatory response in RA remain elusive.

Apoptosis proceeds through two major pathways, an 'intrinsic' pathway that signals through the mitochondria, and an 'extrinsic' pathway that transduces an apoptotic signal following the aggregation of a death receptor to its ligand. The intrinsic pathway is regulated by the Bcl-2 protein family, which are divided into anti-apoptotic (Bcl-2, Bcl-x_L_, Mcl-1, A1/Bfl-1 and Bcl-w) and pro-apoptotic (Bax, Bak, Bad, Bim/Bod, Bok/Mtd, Bik/Blk/Nbk, Bid, Hrk/DP5, Bmf, Noxa, Puma/Bbc3) members [11]. The pro-apoptotic family proteins are divided into two additional groups based on the expression of the Bcl-2-homology (BH 1–4) domain: the multi-BH domain (BH1-3: for example, Bak, Bax) and the BH3-only (for example, Bid, Bim) proteins [12]. Recent studies have suggested that BH3-only proteins are also subdivided into two categories based on their ability to induce apoptosis [13-17]. Bid and Bim are sufficient to sequester anti-apoptotic Bcl-2 family members, induce oligomerization of Bak and Bax, permeabilization of liposomes, and release of cytochrome C [13,14,17]. In contrast, Bad, Bmf, Hrk, Noxa, and Puma are sensitizers for apoptosis since they are able to bind only to the anti-apoptotic Bcl-2 members and require Bid or Bim to induce the death response [13-17]. During the intrinsic apoptotic death, cytochrome C is released into the cytosol from the inner mitochondrial space, where it binds to Apaf1, forming the apoptosome. This complex leads to the activation of initiator pro-caspase 9 [18,19]. Caspase 9 activates caspases 3 and 7 [20], which then induce the downstream degradative events of apoptosis [21]. These events are prevented by the overexpression of Bcl-2/Bcl-x_L _or by the complete ablation of Bax and Bak [22].

The induction of apoptosis mediated by the extrinsic pathway is initiated by binding of death ligands to their receptors, as in the case of Fas ligand (FasL) binding to Fas. Oligomerization of Fas upon FasL binding leads to the recruitment of both FADD and pro-caspase 8 to the carboxyl terminus of Fas [23]. Aggregation or oligomerization of pro-caspase 8 results in its autocatalysis and/or activation, and the induction of the degradative phase of apoptosis through the activation of caspases 3 and 7 [23]. An additional pathway of death receptor-induced cell death may proceed through the mitochondrial pathway by activating the Bcl-2 pro-apoptotic protein Bid [24,25], which is cleaved by caspase 8 following death receptor ligation. Cleaved Bid is targeted to the mitochondria and ultimately results in the induction of apoptosis mediated by the mitochondrial apoptotic pathway [26].

The vast majority of studies in RA have focused on the expression patterns of Bcl-2 family members and death receptor signaling factors in the synovium. Recently, two studies have demonstrated that Fas and Bim are required to limit the inflammatory response in a mouse model of the effector phase of inflammatory arthritis [27,28]. These data suggest that a synergy between the extrinsic and intrinsic apoptotic pathways may be required to prevent or reduce the development of inflammatory arthritis. One potential factor that bridges the two apoptotic pathways is the BH3-only protein Bid. To this end, we examined the impact of deleting Bid (Bid-/-) on the development of inflammatory arthritis in mice. Bid-/- mice show increased ankle swelling accompanied by more articular destruction and a delay in the resolution phase of arthritis. Histological examination of arthritic ankle sections reveals an increase in infiltrating leukocytes, particularly macrophages and neutrophils in Bid-/- mice compared to controls. Furthermore, there are fewer apoptotic cells in Bid-/- mice. Collectively, these data suggest that the decreased apoptosis in Bid-/- mice prolongs the inflammatory phase, leading to enhanced joint destruction and a delay in the resolution phase.

## Materials and methods

### Mice

Bid-/- mice backcrossed for 12 generations onto C57BL/6 background were a kind gift from the late Dr Stanley Korsmeyer (Dana-Farber Cancer Institute, Boston, MA, USA). C57BL/6 mice (congenic control for Bid-/- mice) were purchased from Jackson Laboratory (Bar Harbor, ME, USA). Non-obese diabetes (NOD) mice were purchased from Taconic (Germantown, NY, USA) and the homozygous KRN T-cell receptor transgenic mice (C57BL/6 background) were a kind gift from Drs D Mathis and C Benoist (Harvard Medical School, Boston MA, USA, and the Institute de Gene-tique et de Biologie Moleculaire et Cellulaire, Strasbourg, France). All experiments on mice were approved by the Animal Care and Use Committee at Saint Louis University.

### K/BxN serum transfer-induced arthritis

The KRN T-cell receptor transgenic mouse was crossed with the NOD mouse expressing the A^g7 ^MHC class II allele and all progeny (K/BxN) developed spontaneous arthritis [29,30]. Serum from K/BxN mice may be transferred via intra-peritoneal injection to allogeneic hosts regardless of the genetic background [31]. The host mice develop a transient inflammatory joint disease that lasts for 7 to 14 days. Peripheral blood from seven-week-old K/BxN mice was isolated, and serum were collected and pooled. K/BxN serum (150 μl) was intraperitoneally injected on each flank of 6-week old wild-type (Wt) and Bid-/- mice as previously described [32]. At each time point and prior to euthanasia, the degree of arthritis as indicated by joint swelling was quantified by measuring two perpendicular diameters of the ankles using a caliper (Lange Caliper: Cambridge Scientific Industries, Cambridge, MA, USA). Joint circumference was calculated using the geometric formula of ellipse circumference (2π × v(a^2 ^+ b^2^)/2) as previously described [32]. Following euthanasia, ankle joints were removed and either fixed in 10% neutral buffered formalin for 24 hours, decalcified in EDTA-decalcification buffer for two weeks, embedded in paraffin, and sectioned, or placed in liquid nitrogen, ground into a fine powder by mortar and pestle, digested in protein lysis buffer (150 μM NaCl, 0.5% NP-40, 50 mM Tris, 2 mM EDTA) in the presence of phosphatase and protease inhibitors, homogenized on ice for 20 s, and lysed overnight at 4°C.

### Immunohistochemistry

Paraffin embedded ankle sections were stained with hematoxylin and eosin (H&E) and Safranin O and methyl green. Histopathological scoring was performed as previously described in detail [28,33,34]. A pathologist blinded to the study (GKH) evaluated ankle sections by examining at least 3 sections/ankle and 3 fields/section at 1,000 × magnification. H&E ankle sections were scored on a 0 to 5 scale for inflammation, with 0 = normal, 1 = minimal infiltration, 2 = mild infiltration, 3 = moderate infiltration, 4 = marked infiltration, and 5 = severe infiltration. Bone erosion was scored on a 0 to 5 scale by viewing H&E ankle sections, with 0 = no or normal bone resorption, 1 = small areas of resorption, 2 = more numerous areas of resorption, 3 = obvious resorption, 4 = full thickness defects in the bone without distortion of the profile, 5 = full thickness defects in the bone with distortion of the profile. H&E ankle stained sections were scored on a 0 to 5 scale for pannus formation, with 0 = no pannus formation, 1 = minimal pannus formation, 2 = mild pannus formation, 3 = moderate pannus formation, 4 = marked pannus formation, and 5 = severe pannus formation. Polymorphonuclear (PMN) leukocyte infiltration: 0 = no PMNs, 1 = rare scattered PMNs, 2 = more frequent scattered PMNs, 3 = small clusters of PMNs, 4 = larger clusters of PMNs, and 5 = sheets of PMNs (abscess). Histopathological scoring was conducted on an Olympus BX40 microscope (1,000 ×). Photographs were taken on a Nikon (Tokyo, Japan) microscope equipped with the Nikon digital camera DMX1200.

Macrophages were identified by the expression of F4/80 antigen, a cell surface glycoprotein with homology to the G-protein linked transmembrane 7 hormone receptor family [35]. Previous studies have shown that F4/80 is expressed on all macrophages [36,37] and that macrophages isolated from mice lacking F4/80 do not stain for the F4/80 antigen [38,39]. To stain for F4/80-positivity, antigens were retrieved using the Dako target retrieval solution (Dako, Carpinteria, CA). Following antigen retrieval, sections were blocked in hydrogen peroxide, incubated with anti-F4/80 antibody (Clone BM8; Invitrogen, Carlsbad, CA) or isotype control, and then incubated with secondary biotinylated rabbit anti-rat antibody (Dako). Sections were treated with streptavidin peroxidase conjugate (Dako), color was visualized with diaminobenzidine, and sections were counterstained with hematoxylin. All F4/80 antigen staining was performed on a DAKO autostainer (Dako). Six fields of representative pannus and synovium stained with anti-F4/80 antibody were viewed under oil emersion at 1,000 × magnification, and the number of F4/80 positive cells was counted.

### Immunophenotyping

Peripheral blood was isolated by cardiac sticks from Wt and Bid-/- mice after euthanasia. Nonspecific staining was prevented by incubation with anti-CD16/32 (24G2) antibody (BD Biosciences, San Jose, CA). The blood was incubated with fluorochrome-conjugated antibodies specific for CD3, CD4, CD8, CD19, CD11b, CD45, CD62L, and Gr-1 (BD Biosciences), or isotype controls for 30 minutes at 4°C. After incubation with antibodies, red blood cells were lysed and the samples were fixed by incubation in FACS Lyse (BD Biosciences) for 10 minutes at room temperature. Samples were collected on a BD FACS Calibur at the St Louis University Flow Cytometry Core Facility, and the data were analyzed in FlowJo (TreeStar, Inc. Ashland, OR). Total peripheral blood leukocyte numbers were determined on the automated hematology analyzer ABX Pentra 60.

### ELISA

For detection of mouse CXC chemokine (KC), monocyte chemoattractant protein (MCP-1/CCL2), TNFα, and IL-1β in ankle extracts, sandwich ELISAs were performed according to the manufacturer's instructions (R & D Systems, Minneapolis, MN, USA). The sensitivity of TNFα and MCP-1 ELISAs was 7.8 pg/ml, while the sensitivity of IL-1β and KC ELISAs was 15.6 pg/ml. ELISAs were quantified by absorbance at 450 nm on a microplate reader (BioRad, Hercules, CA, USA). Data obtained using ELISA on ankle extracts (pg/ml) were normalized by the total protein concentration (μg/μl) for each individual ankle extract. The levels of cytokines and chemokines in serum were determined using a Luminex based assay according to manufacturer's specifications (Linco Research, Earth City, MO).

### TUNEL analysis

Paraffin embedded ankle sections (5 μm) were deparaffinized, rehydrated, and permeabilized with 20 μg/ml of proteinase K for 15 minutes. Mouse thymus was used as a positive control for TUNEL (data not shown). TdT enzyme and dUTP conjugated to a fluorescein cocktail were added to sections according to the manufacturer's specifications (*in situ *death detection kit; Roche Biochemical, Indianapolis, IN, USA). Nuclei were stained with Hoechst 33258 (Invitrogen). Slides were mounted with glass coverslips using mounting medium for fluorescence (Kirkegaard and Perry Laboratories Inc., Gaithersburg, MD, USA). Three different areas per joint of TUNEL positive cells were identified at 400 × power. The number of TUNEL positive cells were counted and then divided by the total number of cells in the field as determined by Hoechst staining. The percent of TUNEL positive cells per field was averaged with two other fields identified from different areas of the joint. Photographs were taken on a Nikon microscope equipped with the Nikon digital camera DMX1200.

### Statistical analysis

Results were expressed as the mean ± standard error. Differences between groups were analyzed using Student's *t *test.

## Results

### Bid-/- mice have a delay in the resolution of inflammatory arthritis following transfer of K/BxN serum

**Figure 1:**
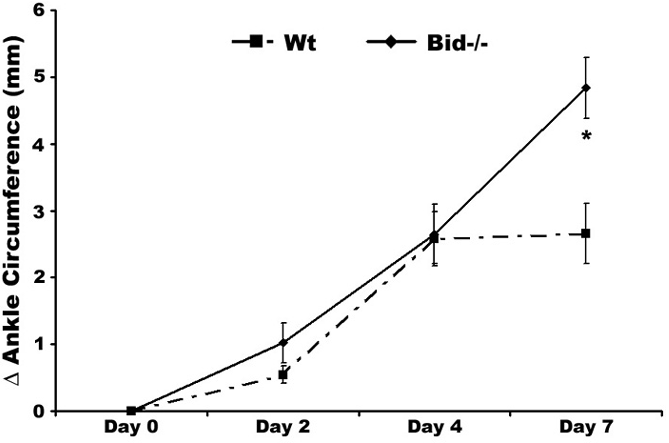
Bid-deficient mice develop a sustained and prolonged edema of the ankles following transfer of K/BxN serum. Pooled serum (300 μl) from K/BxN mice was injected intra-peritoneally (IP) into Bid-/- (*n *= 32) and wild-type (Wt) (*n *= 42) mice. Ankle joints were examined for arthritis by measuring two perpendicular diameters of both joints (anterior-posterior; medio-lateral) by calipers. The change in (Δ) ankle circumference at each time point is defined as the difference between the ankle circumference and the measurement at day 0. values represent the mean ± standard error of ankles/time point, which were compared by Student's *t*-test to Wt mice under parallel conditions. The asterisk denotes *p *< 0.002 compared to Wt under parallel conditions.

Previous studies have implicated the extrinsic and intrinsic apoptotic pathways in preventing or limiting the effector phase of inflammatory arthritis [27,28]. Since the pro-apoptotic protein Bid links the extrinsic to the intrinsic pathway, we examined the affect of inducing experimental inflammatory arthritis in mice lacking Bid (Bid-/- mice). We used the K/BxN serum transfer-induced arthritis model, which is widely used to assess factors that mediate the effector phase of RA. Unlike the collagen-induced arthritis model, the K/BxN model may be used in mice on a C57BL/6 background [31]. This model shares many common features with human RA, including invasion of leukocytes, proliferation of synoviocytes resulting in the thickening of the synovial lining, formation of pannus, and erosion of cartilage and bone [40]. The K/BxN serum transfer model is independent of T and B lymphocytes [30], but requires Fc receptors [41,42] and the alternative pathway of complement [31,42]. There was no difference in edema of the ankle joint in Bid-/- compared to Wt mice at days two and four post-serum transfer as indicated by a change in ankle circumference (Figure 1). However, ankle circumference increased by 2.0-fold (*p *< 0.002) in Bid-/- compared to Wt mice at day seven. There was no change in ankle swelling in Wt mice between days four and seven. These data suggest that the loss of Bid causes impairment in the resolution of K/BxN serum transfer-induced arthritis.

### Arthritic Bid-/- mice display increased histopathological scores

**Figure 2:**
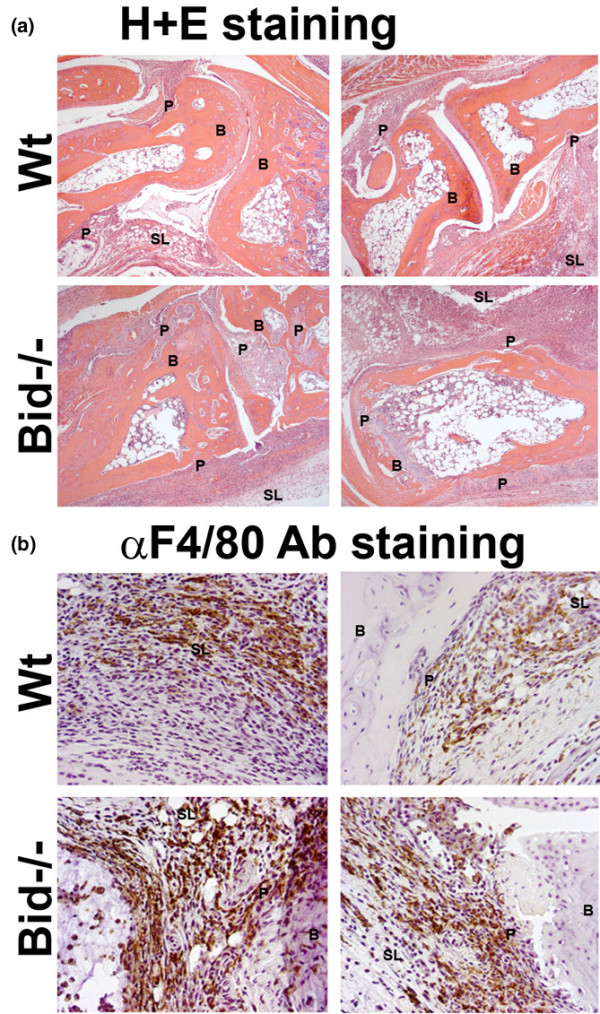
Increased inflammation and destruction of the joint is associated with more macrophages in Bid-/- compared to wild-type (Wt) mice following transfer of serum. Mice (Wt, *n *= 9; Bid-/-, *n *= 7) underwent K/BxN serum transfer as described in Figure 1 and were euthanized at seven days post-serum transfer. Both ankles from each mouse were harvested, fixed, embedded in paraffin, sectioned, and stained with either **(a) **hematoxylin (blue) and eosin (pink) (H&E) or **(b) **F4/80 antigen (macrophage specific marker). Shown are representative photomicrographs of the synovium and pannus formation from Wt and Bid-/- mice. B, bone; SL, synovial lining; P, pannus.

**Figure 3:**
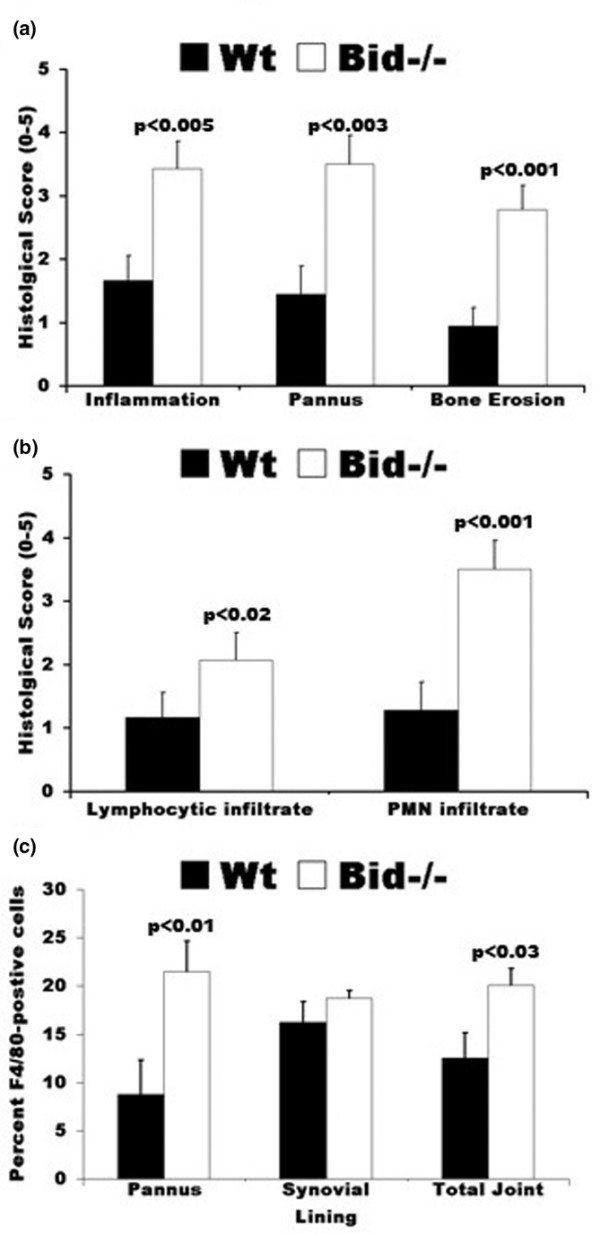
Histological scores of ankle sections from wild-type (Wt) and Bid-/- mice. **(a) **Bid-/- mice have increased inflammation and joint destruction compared to Wt mice. Ankles isolated from mice (Wt, *n *= 9; Bid-/- *n *= 7) were prepared as described in Figure 2. Ankle sections were evaluated and scored by a pathologist blinded to the study as described in the Materials and methods section. Values represent the mean ± standard error of ankles/time point, which were compared by Student's *t*-test. **(b) **Increased numbers of lymphocytes and polymorphonuclear (PMNs) cells in inflamed Bid-/- joints. Ankles were prepared as described above. Values represent the mean ± standard error of ankles/time point, which were compared by Student's *t*-test. **(c) **Arthritic Bid-/- mice have more macrophages in the pannus and in the whole joint. Ankles were examined for F4/80 antigen as described in Materials and methods. The number of positive cells for F4/80 in pannus, synovial lining, and whole joint was determined by a pathologist blinded to the study. Values represent the mean ± standard error of ankles/time point, which were compared by Student's *t*-test.

Ankle sections were examined using a histopathological scoring system to further identify differences in Bid-/- compared to Wt mice following the induction of arthritis. There was a 2.0-fold increase (*p *< 0.005) in inflammation score, a 2.5-fold increase (*p *< 0.003) in pannus formation score, and a 3.0-fold increase in bone erosion score (*p *< 0.001) in Bid-/- compared to Wt ankles (Figures 2a and 3a). No difference in cartilage destruction was detected in Wt and Bid-/- mice (data not shown). Analysis of the cells infiltrating the joints (Figure 3b) showed a 2.7-fold increase (*p *< 0.002) in polymorphonuclear cells and a 2.0-fold increase (*p *< 0.02) in lymphocytes in Bid-/- compared to Wt joints. Bid-/- ankles had a 1.6-fold increase (*p *< 0.03) in the number of macrophages compared to Wt ankles. More specifically, there was a 2.5-fold increase (*p *< 0.02) in the number of macrophages in the pannus of Bid-/- compared to Wt joints. There was no statistical difference in average number of macrophages in the synovial lining in Bid-/- compared to Wt mice. There were no differences in total numbers of lymphocytes, neutrophils, or monocytes circulating in peripheral blood in Wt and Bid-/- mice (Table 1). These data suggest that the increase in the numbers of inflammatory cells in the joints of Bid-/- mice may not be attributed to an elevation in circulating leukocytes. Since the K/BxN serum transfer model has been shown to be independent of T and B cells [30], these data suggest that the impairment in the resolution of arthritis in Bid-/- mice may be due to an inability to clear infiltrating leukocytes, specifically neutrophils and macrophages.

**Table 1:** Wt and Bid-/- mice have similar numbers of leukocyte subpopulations in peripheral blood

	CD19+	CD3+	CD3+	CD3+	CD11b+	CD11b+	CD11b+
			CD4+	CD8+	Gr-1-	Gr-1+	Gr-1++
					CD62L -	CD62L+	

Wt (*n *= 20)	46.5 ± 0.8	22.5 ± 0.6	12.1 ± 0.4	7.4 ± 0.3	3.5 ± 0.2	2.6 ± 0.2	6.2 ± 0.8
Bid-/- (*n *= 15)	40.2 ± 3.8	21.1 ± 1.4	12.2 ± 1.0	8.5 ± 1.1	4.1 ± 0.7	2.6 ± 0.6	5.7 ± 0.5

Quantitative analysis of peripheral blood leukocyte numbers (expressed as 1 × E5/ml) in blood isolated from wild-type (Wt) and Bid-/- mice. All data expressed as means ± standard error.

### Expression of pro-inflammatory factors is similar in Wt and Bid-/- mice following serum transfer

**Figure 4:**
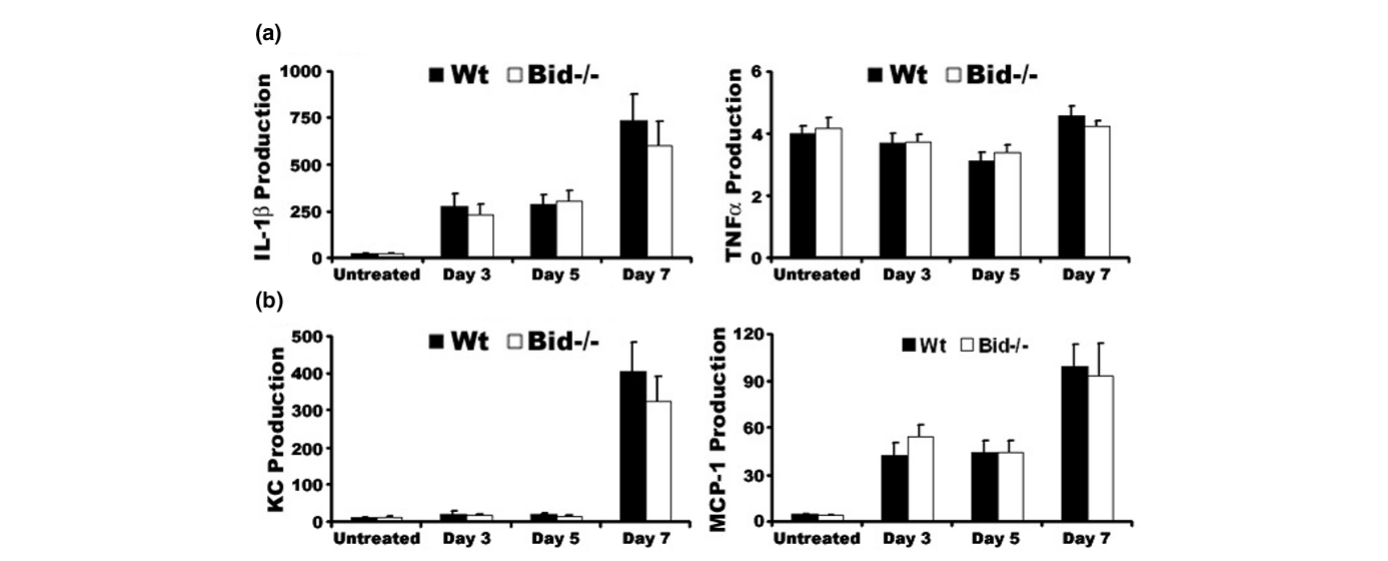
Loss of Bid does not alter the cytokine and chemokine milieu of the joint. **(a) **Pro-inflammatory cytokine production in ankle joints following transfer of K/BxN serum. Untreated wild-type (Wt) and Bid-/- mice were euthanized at three, five, or seven days post-serum transfer. Ankles from each mouse (days 3, *n *= 6 (Wt) and n= 8 (Bid-/-); day 5, *n *= 10; day 7, *n *= 12 (Wt) and *n *= 8 (Bid-/-)) were isolated, snap frozen, ground into a fine powder, lysed, and examined for production of tumor necrosis factor (TNF)α and IL-1β using sandwich ELISAs. **(b) **Chemokine production in ankle joints following transfer of K/BxN serum. Ankles lysates as described above were examined for production of CXC chemokine (KC) and monocyte chemoattractant protein (MCP)-1 using ELISA. Data are shown as μg/μl per joint. Values represent the mean ± standard error, which were compared by Student's *t*-test.

The cytokine and chemokine milieu of the joint is necessary for the initiation and the perpetuation of inflammatory arthritis. Previous studies have shown that lpr and Bim-/- mice display increased levels of pro-inflammatory factors in the joint and in serum [27,28]. There were no differences in TNFα, IL-1β, KC, or MCP-1 levels in Bid-/- and Wt untreated ankle joints and in ankle joints isolated at days 3, 5, or 7 post transfer of serum (Figure 4). However, there was a 2.0-fold increase in circulating levels of IL-1β (*p *> 0.09) and KC (*p *< 0.01) in Bid-/- compared to Wt serum at day 3 post-serum transfer (Table 2). There were no differences in untreated serum samples from Wt and Bid-/- mice (Table 2). These data suggest that while the local inflammatory milieu remains similar in Bid-/- and Wt mice, the systemic levels of IL-1β and KC are increased. These elevated levels of IL-1β and/or KC may lead to the increased numbers of neutrophils and macrophages in Bid-/- mice.

**Table 2:** Arthritic Bid-/- mice have elevated levels of serum KC and IL-1β

	Day 0		Day 3		Day 5	
			
	Wt	Bid-/-	Wt	Bid-/-	Wt	Bid-/-
G-CSF	56.0 ± 42.0	45.6 ± 9.3	454.1 ± 92.5	488.1 ± 84.3	869.9 ± 366.0	496.7 ± 91.2
GM-CSF	UD	UD	9.6 ± 9.6	115.7 ± 54.3	58.8 ± 58.8	20.7 ± 20.7
IL-10	UD	UD	UD	61.6 ± 45.3	111.7 ± 60.3	16.7 ± 16.7
IL-12 (p70)	UD	9.2 ± 9.2	UD	22.1 ± 10.4	29.4 ± 20.8	UD
IL-1β	3.7 ± 1.3	3.5 ± 1.5	24.3 ± 2.6	43.6 ± 8.8 (*p *< 0.09)	44.72 ± 4.2	34.1 ± 3.0
IL-6	2.4 ± 1.6	0.6 ± 0.6	165.3 ± 31.5	240.6 ± 38.2	207.2 ± 53.2	166.9 ± 19.9
KC	7.2 ± 3.5	3.9 ± 2.5	53.8 ± 7.9	108.2 ± 13.2 (*p *< 0.01)	113.5 ± 25.6	75.2 ± 8.1
MCP-1	1.2 ± 1.2	4.8 ± 4.8	1.0 ± 0.2	3.2 ± 1.9	2.8 ± 0.8	2.2 ± 0.1
MIP1α	7.5 ± 3.0	60.5 ± 40.5	100.9 ± 28.2	161.5 ± 27.3	186.9 ± 17.3	191.0 ± 32.1
RANTES	1.2 ± 0.8	24.6 ± 24.6	5.8 ± 0.6	6.9 ± 0.9	9.7 ± 2.0	6.6 ± 0.7
TNFα	3.0± 0.4	3.1 ± 0.4	7.7 ± 2.6	13.0 ± 6.4	15.6 ± 2.3	15.2 ± 0.9

Wild-type (Wt) (*n *= 5/tp) and Bid-/- (*n *= 5/tp) mice were euthanized at three or five days post-serum transfer. Peripheral blood was isolated by cardiac stick, and serum was separated by centrifugation and examined for production of the indicated cytokine or chemokine (pg/ml) using a Luminex based assay. Values represent the mean ± standard error, which were compared by Student's *t*-test. G-CSF, granulocyte colony stimulating factor; GM-CSF, granulocyte-macrophage colony stimulating factor; KC, CXC chemokine; MCP, monocyte chemoattractant protein; MIP1α, Macrophage inflammatory protein 1α; RANTES, regulated upon activation, normal T-cell expressed, and secreted; TNF, tumor necrosis factor; UD, undetectable.

### Bid-deficient mice have a decrease in apoptotic cells in the joint following serum transfer

**Figure 5:**
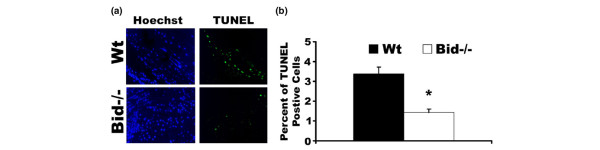
Bid-/- mice have fewer TUNEL positive cells following serum transfer. **(a) **Decreased numbers of apoptotic cells in Bid-/- ankle joints following transfer of K/BxN serum. Ankle sections from Bid-/- mice and wild-type (Wt) mice as described in Figure 2 were stained with Hoechst (blue) and TUNEL (green) as detailed in the Materials and methods section. Shown are representative photomicrographs (200 × magnification) of ankle joints (*n *= 14 for Bid-/- and *n *= 18 for Wt) isolated seven days post-serum transfer. **(b) **Percentage of TUNEL-positive cells in the joint. Ankle joints as described above were stained with TUNEL and Hoechst. Three areas of TUNEL positive cells were examined (400 × magnification) and percentages of TUNEL-positive cells were determined as described in Materials and methods. The values represent the mean ± standard error, which were compared by Student's *t*-test. The asterisk denotes *p *< 0.001 compared to Wt under parallel conditions.

To assess whether the increased inflammation and destruction of the joints in Bid-/- mice was due to deficiencies in apoptosis, we examined the joints for apoptotic cells using TUNEL. The apoptotic cells in the joints of Wt mice were mainly located near the bone and cartilage junction, similar to previous studies [28]. At seven days post-serum transfer, Wt ankles had a 2.4-fold (*p *< 0.001) increase in TUNEL positive cells compared to Bid-/- joints (Figure 5). These data suggest that the failure to resolve the arthritis in Bid-/- mice may be due to an inability to delete the autoreactive cells in the joint.

## Discussion

Over the past years the notion that a lack of apoptosis contributes to the increase in synovial lining in RA patients has gained momentum [4]. Early studies on RA synovial tissue failed to find significant numbers of cells positive for TUNEL, membrane blebbing, or condensed chromatin [43]. Furthermore, various factors known to be involved in apoptosis have been examined in these tissues, including TRAIL [44], Fas [45], Flip [45], Bcl-2 [46], Bcl-x_L _[47], Bax [48], and Puma [49]. Recently, we have shown that mice lacking Fas or Bim, two proteins that function as pro-apoptotic proteins in the extrinsic and intrinsic pathways, respectively, display a worse form of K/BxN serum transfer-induced arthritis [27,28]. These are the first studies to utilize mouse genetics to study the role of apoptotic molecules in inflammatory arthritis. Since Fas and Bim function in two different apoptotic pathways, we hypothesized that Bid, which connects the extrinsic to the intrinsic apoptotic pathway, will play a crucial role in the development of inflammatory arthritis.

Here, we show that Bid-/- mice fail to resolve K/BxN serum transfer-induced arthritis. Bid-/- mice have increased numbers of macrophages and neutrophils, two essential cell types for the K/BxN serum transfer-induced arthritis model, and fewer apoptotic cells in joints, similar to lpr and Bim-/- mice [27,28]. These data suggest that the extrinsic and intrinsic pathways may synergize to limit the effector phase of arthritis by inducing apoptosis. They also suggest that Bcl-2 pro-apoptotic members are central for the elimination of autoreactive cells. While both Bid and Bim function as activators in apoptosis, Bid must be cleaved into its active form to be effective at inducing apoptosis. Thus, it is possible that Bim-induced apoptosis in synoviocytes or infiltrating leukocytes may lead to the activation of Bid through a positive feedback loop. Alternatively, death receptor-induced apoptosis, including Fas and TRAIL, may lead to the activation of Bid through caspase 8-mediated cleavage of Bid. While we have shown that Fas-mutant mice develop a more severe form of arthritis [27], there was no statistical difference in the number of apoptotic cells in Wt and lpr mice at seven days post-serum transfer (data not shown). Although these data suggest that Fas-mediated suppression of arthritis may be independent of Bid, the apoptotic cells in lpr mice may be phagocytosed at a slower rate, which occurs in mice that develop lupus-like disease. Thus, lpr mice may also have less apoptotic cells over time due to an inability to activate Bid and the apoptotic cascade. Future studies will be necessary to examine whether there is a deficiency in the activation of Bid in lpr or Bim-/- mice.

A deficiency in pro-apoptotic proteins leads to increased numbers of cells in the synovium through enhanced proliferation, lack of death, or increased migration of infiltrating leukocytes. Bid-/- mice only show a difference in edema of the ankle at day 7, while Bim-/- mice display a difference in ankle swelling as early as day 2 post-serum transfer. Mice lacking Bid or Bim exhibit increased numbers of neutrophils and macrophages in the joint and fewer apoptotic cells. In contrast to Bim-/- or lpr mice [27,28], the local levels of pro-inflammatory cytokines and chemokines and the number of circulating leukocytes are similar in Wt and Bid-/- mice. These data suggest that Fas and Bim modulate the production of pro-inflammatory factors and numbers of leukocytes independently of each other and independent of apoptosis in the joint. Recently, a study showed that Bim-/- dendritic cells induced a hyperactivation of T-cells compared to Wt cells [50]. These data suggest that the loss of Bim or Fas may lead to enhanced activation of effector cells involved in the pathogenesis of RA. Future studies will be necessary to identify the mechanism of the enhanced production of pro-inflammatory factors and maintenance of leukocyte homeostasis in lpr and Bim-/- mice compared to Bid-/- mice.

Patients with RA have increased numbers of monocytes, particularly inflammatory monocytes, circulating in peripheral blood [51-53] and they have elevated numbers of macrophages in the joint [2,3]. Macrophage numbers are associated with articular destruction in RA patients [2,3] and these macrophages are highly activated and contribute directly to synovial inflammation and destruction of cartilage and bone [54,55]. Macrophages are one of the central producers of IL-1β and TNFα, two essential pro-inflammatory cytokines required for the progression of RA because they are capable of inducing other pro-inflammatory cytokines and activating matrix metalloproteinases in autocrine and paracrine fashions [56]. Inhibitors of IL-1β and TNFα cause a reduction in synovial inflammation, bone destruction, and macrophage infiltration in RA patients [57-59]. Recently, suppression of TNFα by administration of soluble TNFα receptor or anti-TNFα antibody has been shown to induce apoptosis in macrophages but not in lymphocytes isolated from the joint [1]. Furthermore, monocytes and macrophages are required for the development of collagen-induced arthritis, IL-1/mBSA-induced arthritis, and K/BxN serum transfer-induced arthritis [60-62]. Recently, Bid has been shown to be essential for maintaining macrophage homeostasis in mice [63]. Mice that lack Bid develop myeloid tumors over time and display decreased survival rates due to these tumors [63]. Thus, in the RA joint, Bid may be suppressed, thereby allowing for increased numbers of macrophages. However, therapies such as TNFα antagonists may allow the activation of Bid and induce apoptosis of macrophages.

## Conclusion

These studies document a synergistic role for the extrinsic and intrinsic apoptotic pathways in inflammatory arthritis. Bid, which is the focal point between these two apoptotic pathways, is essential for the resolution phase of K/BxN serum transfer-induced arthritis. Mice lacking Bid display increased arthritis associated with more inflammation, pannus formation, bone destruction, and infiltrating leukocytes. Furthermore, there are fewer apoptotic cells in the joints of Bid-/- compared to Wt mice. These data document that Bid is a crucial intermediary for the apoptotic pathways in the development of inflammatory arthritis.

## Abbreviations

BH = Bcl-2-homology; Wt = wild-type; ELISA = enzyme-linked immunosorbent assay; FasL = Fas ligand; H&E = hematoxylin and eosin; IL = interleukin; KC = CXC chemokine; MCP = monocyte chemoattractant protein; PMN = polymorphonuclear; RA = rheumatoid arthritis; TNF = tumor necrosis factor; TUNEL = terminal transferase dUTP nick end labeling.

## Competing interests

The authors declare that they have no competing interests.

## Authors' contributions

JCS prepared the arthritis model, and performed the ELISAs and apoptosis assays. JH performed all flow cytometry work. EB assisted with all measurements for arthritis. GKH was the pathologist who analyzed the tissue sections. HP supervised all the work and wrote the majority of the manuscript.
